# Upregulated microRNA-106a Promotes Porcine Preadipocyte Proliferation and Differentiation by Targeting Different Genes

**DOI:** 10.3390/genes10100805

**Published:** 2019-10-14

**Authors:** Kuilong Huang, Xin’e Shi, Jie Wang, Ying Yao, Ying Peng, Xiaochang Chen, Xiao Li, Gongshe Yang

**Affiliations:** Key Laboratory of Animal Genetics, Breeding and Reproduction of Shaanxi Province, Laboratory of Animal Fat Deposition and Muscle Development, College of Animal Science and Technology, Northwest A & F University, Yangling 712100, Shanxi, China; hklgood@163.com (K.H.); xineshi@nwafu.edu.cn (X.S.); wangjie93@nwafu.edu.cn (J.W.); yaoying@nwafu.edu.cn (Y.Y.); py9101@163.com (Y.P.); cxca_01@nwsuaf.edu.cn (X.C.); nice.lixiao@gmail.com (X.L.)

**Keywords:** miR-106a, proliferation, differentiation, porcine preadipocytes, p21, BAMBI

## Abstract

Adipose tissue is one of the main organs for the energy storage and supply of organisms. Adipose deposition and metabolism are controlled by a cascade of transcription factors and epigenetic regulatory mechanisms. Previous studies have also shown that miR-106a plays a considerable role in the development of organisms. The regulatory mechanism of miR-106a on porcine preadipocytes is still not clear. In this study, preadipocytes were isolated from the neck subcutaneous deposits of 3–5-day old Chinese native Guanzhong black pigs using 5-ethynyl-20-deoxyuridine (EdU) staining and a CCK-8 assay to detect the number of proliferous cells and real-time qPCR (RT-qPCR) and western blot analysis to detect gene expression, as well as Oil Red O and BODIPY staining dye lipid droplets and flow cytometry (FCM) to detect cell cycles. We also used the double luciferase method to detect the relative luciferase activities. Upregulated miR-106a increased the number of proliferous cells and enhanced the expression of cell proliferation-related genes in porcine adipocytes. The double luciferase reporter vector confirmed that *p21* was a target gene of miR-106a in the cell proliferation phase. miR-106a upregulation increased the number of lipid droplets and the expression of lipogenic genes and directly targeted *BMP and activin membrane-bound inhibitor (BAMBI)* in the process of differentiation. Our results indicated that miR-106a promotes porcine preadipocyte proliferation and differentiation by targeting *p21* and *BAMBI*.

## 1. Introduction

Adipose tissue is an important endocrine organ of the body, which not only affects the health of the body, but also affects the meat quality of livestock. White adipose tissue (WAT) dysfunction underpins the association of obesity with the development of cardiovascular disease, hepatic steatosis, and cancers [1,2,3,4]. In mammals, WAT mainly includes subcutaneous fat, visceral fat, and intramuscular fat. Moreover, studies have revealed that backfat thickness is highly associated with intramuscular fat content, meat color, tenderness, drip loss, cooking loss, and collagen proteins [5]. Adipogenesis is required for the maintenance of adipose tissue functions. Therefore, the study of the mechanism of mammalian fat deposition is of great significance. In this study, we isolated porcine subcutaneous pre-adipocytes as experimental materials to study the molecular mechanisms of preadipocyte adipogenesis. miRNAs are small RNAs of 18–22 nt in length that degrade gene mRNA or inhibit gene translation by targeting the 3’UTR of the gene [6]. Research showed that miRNAs play crucial roles during fat deposition [7,8,9]. Moreover, dicer plays an essential role in the formation and differentiation of white adipose tissue [10]. Emerging evidence has found that miRNAs have important regulatory effects on the physiological processes of adipocytes, such as their proliferation, differentiation, and apoptosis [11,12,13]. Meanwhile, accumulating evidence also indicates that miRNAs play an important role in the development of porcine adipocytes [14,15,16,17].

The sequence of the miR-17-92 gene cluster is highly conserved in all vertebrates. Meanwhile, the miR-17-92 gene cluster is highly expressed in a variety of solid tumors, called “oncogenes” [18,19,20,21]. In mammals, the miR-17-92 gene cluster has two paralogs: the miR-106a-363 and miR-106b-25 gene clusters, wherein the miR-106a-363 gene cluster is located on the X chromosome, including miR-106a, miR-18b, miR-20b, miR-19b-2, miR-92-2, and miR-363. Some research groups have confirmed that, in vivo, the miR-17-92 gene cluster may participate in and maintain the differentiation of pluripotent cells in the early development of embryos and play an important regulatory role in the development and formation of tissues and organs [22,23,24,25]. In addition, the miR-17-92 family is also closely related to the TGF-β signaling pathway [26,27]. Previous studies have also shown that miR-106a plays an important role in the development of organisms as an important member of the miR-17-92 gene cluster [28,29]. More importantly, studies have shown that miR-106a could promote human adipose-derived mesenchymal stem cell adipogenesis and inhibit osteogenesis [30]. However, the regulatory mechanism of miR-106a on porcine preadipocytes is still unclear. Therefore, this study tried to elucidate the mechanism of miR-106a on proliferation and differentiation of porcine preadipocytes.

In the present study, we illustrate that upregulated miR-106a can promote porcine preadipocyte proliferation by targeting *p21*. In addition, overexpression of miR-106a can induce adipogenesis in porcine preadipocytes by targeting *BMP and activin membrane-bound inhibitor* (*BAMBI*). Our findings revealed a potential mechanism regulated by miR-106a, p21, or *BAMBI* acting in the development of preadipocytes. These results provide a theoretical foundation for increasing pork production and a potential therapeutic target against metabolic diseases induced by obesity.

## 2. Materials and Methods 

### 2.1. Isolation and Culture of Porcine Preadipocytes

Porcine preadipocytes were isolated from the subcutaneous fat of 3–5-day old piglets as in previous descriptions of the laboratory procedures [31]. The tissue was washed 3 times with phosphate buffer saline (PBS), and then the tissue was cut into a size of 1–2 mm^3^. Then, the porcine preadipocytes were poured into 1 mg/mL of type I collagenase. The collagenase was digested in 37 °C water for 1–1.5 h. Digestion was terminated by adding an equal volume of medium containing 10% FBS. The digested liquid was filtered through a 70 μm filter and then centrifuged at 421 rcf for 10 min and washed 3 times with a serum-free medium. It was then inoculated into a large dish. For adipogenic differentiation, when the cells reach confluence, the adipogenic inducer cocktail DMI (Dulbecco’s modified Eagle medium (DMEM)/F12 with 10% fetal calf serum (FBS) and the addition of 0.5 mmol/L 3-isobutyl-1-methylanxthin (IBMX), 1 μmol/L dexamethasone (Dex), 5 μg/mL insulin) was added into the growth medium. After 2 days, the medium was changed into the growth medium supplemented with 5 µg/mL insulin for 6–8 days until cell maturation. Porcine sample handling accorded with the ethics committee of Northwest A & F University (Yangling, China) (14-233, 10 December 2014), ethic approval number NWAFU-314020038.

### 2.2. Transfection of miRNA Agomir

miR-106a negative control (NC) and agomir were purchased from Genepharma (Shanghai, China) and were transfected into the cells by X-tremeGENE HP DNA Transfection Reagent (Roche, Mannheim, Germany). The sequence of the miRNAs was:

Ssc-NC sense: 5-UUCUCCGAACGUGUCACGUTT-3;
Ssc-antisense: 5-ACGUGACACGUUCGGAGAATT-3;
Ssc-miR-106a sense: 5-AAAAGUGCUUACAGUGCAGGUAGC-3;
Ssc-antisense: 5-UACCUGCACUGUAAGCACUUUUUU-3.

### 2.3. RNA Extractions and RT-qPCR

Total RNA was extracted with a TRIzol reagent (TaKaRa, Otsu, Japan). RNA reverse transcription PCR was carried out using reverse transcription kits (TaKaRa). We used Applied Biosystems (stepOnePlus, Thermo Fisher Scientific, USA) and a SYBR green kit (Vazyme, Nanjing, China) to complete the qPCR reactions. Relative gene expression was analyzed using the 2^−ΔΔct^ method. The expressions of all genes were normalized to β-actin. The U6 small RNA was the internal reference when examining the level of miR-106a. The primer sequences used for real-time qPCR (RT-qPCR) analyses are listed in Table 1.

### 2.4. Western Blot Analysis

Cells were split by radio immunoprecipitation assay (RIPA) buffer (Beyotime, Shanghai, China) and supplemented with a 100 × protease inhibitor (Pierce, Rockford, IL, USA). The protein was separated by sodium dodecyl sulfate (SDS) polyacrylamide gel electrophoresis, and then was transferred to a polyvinylidene Fluoride (PVDF) membrane (Millipore, Boston, MA, USA). Next, the protein was incubated with the corresponding primary antibody and secondary antibody. Antibodies included adipose triglyceride lipase (ATGL) (1:500, Cell Signaling, Boston, MA, USA cat. no. 2138), peroxisome proliferator-activated receptor γ (PPARγ) (cat. no. sc-7196), fatty acid binding proteins (aP2) (cat. no. sc-271529), fatty acid synthase (FAS) (cat. no. sc-21730), cyclin B (cat. no. sc-752), cyclin E (cat. no. sc-481), proliferating cell nuclear antigen (PCNA) (cat. no. sc-56), p21 (cat. no. sc-6246) (1:500 Santa Cruz, Dallas, TX, USA), cyclin D (1:500 BOSTER, China cat. no. BA0770), β-actin (1:500 Sungene Biotech, Tianjin, China), and BAMBI (1:500 Thermo Fisher Scientific, Waltham, MA, USA cat. no. PA5-38027).

### 2.5. 5-Ethynyl-20-Deoxyuridine (EdU) Staining

The proliferation of porcine preadipocytes was detected by the Click-iTEdUAlexa Fluor 594 Imaging Kit (Invitrogen, Carlsbad, CA, USA). Cell nuclei were stained with Hoechst (Beyotime, Shanghai, China) for 15 min. Finally, cells were examined using a Nikon TE 2000 microscope (Nikon, Tokyo, Japan). Cells in each field of view were then counted and analyzed by the Photoshop software EdU (positive cells/nuclei).

### 2.6. Cell Counting Kit-8 (CCK-8)

Porcine preadipocytes were seeded in 96-well plates. After transfection with NC/agomir for 48 h, 10 μL of CCK-8 reagent (Vazyme) were added per dish (lucifugal operation). Then, it was incubated 4 h at 37 °C in a cell incubator. Last, the medium was measured for the absorbance at 450 nm.

### 2.7. Flow Cytometry

Flow cytometry was done using a FACScan argon laser cytometer (Becton Dickinson, Franklin Lakes, NJ, USA), and the fraction of cells in each phase of the cell cycle was determined using the cell cycle analysis platform in the FlowJo software (Tree Star Inc., Ashland, OR, USA).

### 2.8. Luciferase Reporter Assay

The cDNA of p21 and that containing the binding site of miR-106a was amplified by reverse transcription PCR (RT-PCR) from the total RNA extracted from porcine preadipocytes and cut with primers tagged with the XhoI and KpnI cutting sites (TaKaRa). The wild-type or mutated 3`-UTR fragment was cloned into psiCHECKTM-2 Vector (Promega, Madison, WI, USA) at the 3`-end of the Renilla gene. More than 2.0 × 10^4^ porcine preadipocytes were plated in 24-well plates 24 h before transfection. Cells were cotransfected with 200 ng of vector constructs and 100 nmol/L of miR-106a NC/agomir per well by the X-tremeGENE HP DNA Transfection Reagent (Roche, Basel, Switzerland). After 48 h of transfection, luciferase activity was measured by DualGlo Luciferase Assay System (Promega, Madison, WI, USA). 

### 2.9. BODIPY Staining of Lipid Droplets

On the 8th day of porcine adipocyte differentiation, the cells were washed with PBS three times and fixed with 4% paraformaldehyde at 37 °C for 30 min. Subsequently, cells were incubated with BODIPY (Invitrogen) (stock concentration 1 mg/mL, working solution 1:1000 dilution) for 30 min. Next, the cells were washed three times with PBS and then the nuclei were stained with 4,6-diamino-2-phenyl indole (DAPI) (Invitrogen) for 15 min and washed with PBS 3 times; finally, images were captured using a fluorescence microscope (Nikon, Tokyo, Japan).

### 2.10. Oil Red O Staining and Dye Extraction Analysis

After adding DM, porcine preadipocytes were cultured for 8 days. Cells were fixed with 4% paraformaldehyde for 30 min at room temperature. Afterwards, the fixed cells were washed and stained with 1% filtered Oil Red O for 30 min. Subsequently, cells were washed and observed with phase-contrast microscopy (IS-Elements software, Nikon Eclipse, Tokyo, Japan). Finally, isopropanol was added into the cells for 20 min, and a quantitative analysis was performed using a colorimetric spectrophotometer.

### 2.11. Bioinformatics Method

We performed a bioinformatics analysis using TargetScanmiRBase and miRTarBase. 

Many thousands of potential target genes were predicted. The common target gene associated with myogenes was predicted by at least 3 programs. Meanwhile, we applied KOBAS 3.0 to complete the gene ontology (GO) analysis and Kyoto encyclopedia of genes and genomes (KEGG) analysis.

### 2.12. Statistical Analysis

All quantitative data were performed at least 3 times independently. Results are represented as the mean ± SEM. GraphPad Prism 6 was utilized to graph the results. One-way analysis of variance (ANOVA) in the SPSS 17 software (SPSS Inc., Chicago, IL, USA) was used to perform variance analysis and significance tests. The statistical significance is represented as follows: * *p* < 0.05, ** *p* < 0.01.

## 3. Results

### 3.1. miR-106a is Widely Expressed in Various Porcine Tissues and is Closely Related to The Cell Cycle and Adipose Metabolism

**Figure 1:**
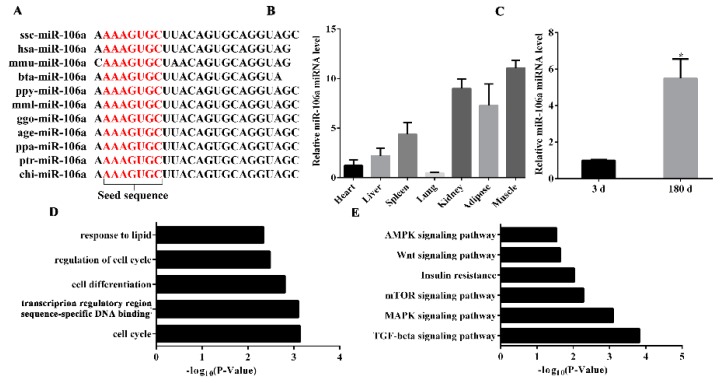
The expression level of miR-106a in different porcine tissues and a bioinformatics analysis of miR-106a. (**A**) The seed sequence of miR-106a is highly conserved across species; (**B**) miR-106a expression in seven different tissues of 180-day old Guanzhong black pigs; (C) miR-106a expression in the adipose tissue of 3-day and 180-day Guanzhong black pigs; (**D**) gene ontology (GO) term analysis of the miR-106a targets; (**E**) Kyoto encyclopedia of genes and genomes (KEGG) pathway analysis of the miR-106a targets. Data are representative of the mean ± SEM; n = 4. * *p* < 0.05, ** *p* < 0.01.

The miR-106a seed sequence is highly conserved in various species, including *Sus* *scrofa*, *Homo sapiens*, *Mus musculus*, and *Bos taurus* suggesting its important function in biological processes (Figure 1A). To clarify the role of miR-106a in porcine tissue, we extracted RNA from various tissues of 3–7 day old Guanzhong black pigs and measured the expression of miR-106a by RT-qPCR. The data showed that miR-106a is widely expressed in various tissues of pigs, but its expression in adipose tissue, muscles, and kidneys is significantly higher than its expression in other tissues (Figure 1B). In addition, we detected the expression level of miR-106a in the adipose tissue of Guanzhong black pigs at 3 days and 180 days. It was found that the expression level of miR-106a was significantly increased at 180 days (*p* < 0.05) (Figure 1C). These results suggest that miR-106a may take part in the adipocyte differentiation process. To investigate the function of miR-106a, the target genes of miR-106a were analyzed by GO and KEGG. The GO results showed that miR-106a was closely related to the cell cycle and lipid metabolism (Figure 1D). The TGF-β pathway was the most likely pathway used by miR-106a by KEGG (Figure 1E).

### 3.2. Upregulated miR-106a Promotes the Proliferation of Porcine Preadipocytes

**Figure 2:**
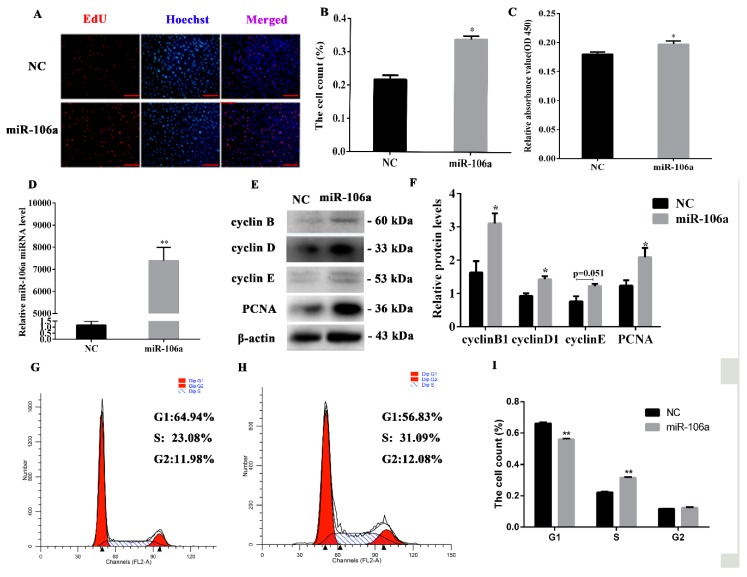
The overexpression of miR-106a promotes porcine preadipocyte proliferation. The miR-106a agomir or negative control (NC) were transfected into cells, and cells were harvested 48 h after transfection. (**A**,**B**) 5-Ethynyl-20-deoxyuridine (EdU) staining assayed the number of proliferous cells; positive cells were stained by EdU (red) and total cell nucleus was stained with Hoechst (blue); (**C**) the cell count was measured by cell count kit-8 (CCK-8), and the results illustrated the absorbance value at 450 nm, after incubation with 10% CCK-8 solution for 4 hours; (**D**) the overexpression efficiency of miR-106a after transfection with the miR-106a agomir compared with NC; (**E**) western blot analysis of cell cycle genes; (**F**) quantification of the western blot analysis of cyclin B, cyclin D, cyclin E and proliferating cell nuclear antigen (PCNA); (**G**,**H**) cell cycle analysis was performed by a flow cytometer; (**I**) the statistical results of cell cycle analysis. Data are representative of the mean ± SEM of three independent experiments; scale bar: 100 μm; * *p* < 0.05, ** *p* < 0.01.

To elucidate the potential role of miR-106a in adipogenesis, an miR-106a agomir or negative control (NC) was transfected into porcine preadipocytes. After 48 h, compared with the control group, the result of the EdU staining showed that the proportion of positive cells was increased dramatically in cells transfected with miR-106a agomir (Figure 2A,B). Moreover, the CCK-8 experiment showed that the 450 nm optical density (OD) value was increased (Figure 2C). miR-106 expression was outstandingly upregulated (*p* < 0.05) (Figure 2D). Cell cycle markers, including cyclinB, cyclinD, cyclinE, and cell proliferation-related gene PCNA, were up-regulated at the protein levels (Figure 2E,F). In addition, the results of the flow cytometry analysis indicated that miR-106a led to cell-cycle acceleration, with an increase in the fraction of S-phase cells and a declining G1 phase proportion (Figure 2G). Taken together, these results suggest that upregulated miR-106a promotes the proliferation of porcine preadipocytes, indicating that miR-106a may be a positive adipogenic regulator.

### 3.3. miR-106a Can Target p21 Instead of BAMBI in Proliferative Phase

**Figure 3:**
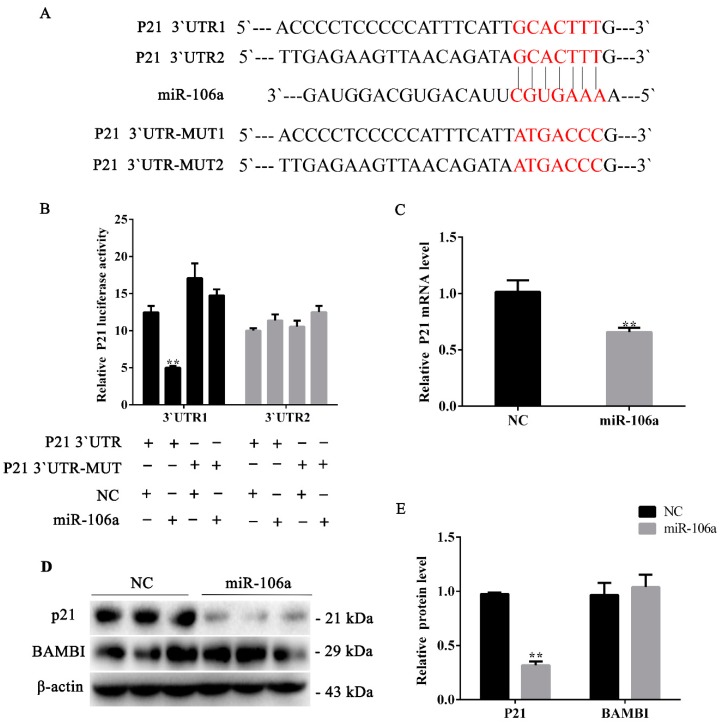
miR-106a can target *cyclin-dependent kinase inhibitor 1A (p21)* instead of BAMBI in the proliferative phase. (**A**) The target site of miR-106a within the porcine *p21* mRNA 3′UTR and mutational site of *p21* 3′UTR; (**B**) a dual-luciferase assay was performed by com-transfection of the miR-106a agomir and wild-type vectors or mutant vectors; relative luciferase activity was represented by Renilla luciferase/firefly luciferase (RLUC/FLUC); (**C**) the relative *p21* mRNA expression levels after treatment with the miR-106a agomir; (**D**) western blot analysis of p21 and BAMBI protein expression after treatment with the miR-106a agomir; (**E**) the quantification of p21 and BAMBI protein expression levels. Data are representative of the mean ± SEM of three independent experiments. * *p* < 0.05, ** *p* < 0.01.

Previous experiments showed that miR-106a can promote porcine preadipocyte proliferation (Figure 2). In order to identify the target gene by which miR-106a promotes adipocyte proliferation, we performed prediction of the target genes by using TargetScan7.2 and miRTarBase. We selected *p21* as its target gene from a number of predicted candidate genes, and the psiCHECKTM-2 vector and the mutation vector were constructed for the two predicted target sites at the 3`UTR of p21 (Figure 3A). 

**Figure 4:**
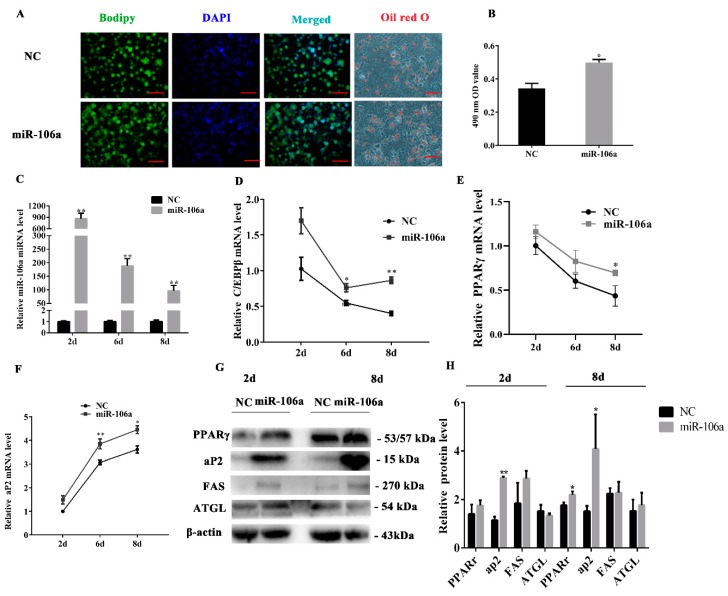
The overexpression of miR-106a accelerates porcine preadipocyte differentiation. The miR-106a agomir or NC were transfected into cells at 80% density at 50 nmol/L; (**A**) boron dipyrromethene (BODIPY) staining was performed in cells on the 8th day of differentiation, the lipid droplets were stained with BODIPY (green) and the cell nucleus was stained with DAPI (blue). Oil Red O staining was performed at day 6 after adipogenic differentiation; (**B**) triglycerides content was measured by spectrophotometric analysis at 490 nm; (**C**) the overexpression efficiency of miR-106a after transfection with the miR-106a agomir; (**D**–**F**) real-time qPCR (RT-Qpcr) was used to detect adipogenesis genes, CCAAT/enhancer-binding protein β (C/EBPβ), peroxisome proliferator- activated receptor γ (PPARγ), and fatty acid binding proteins (aP2) on the 2nd day, 6th day, and 8th day after adipogenic induction; (**G**) western blot analysis of the adipogenic genes’ protein expression on the 2nd day and 8th day after adipogenic induction; (**H**) the quantification of adipogenic genes protein expression levels. Data are representative of the mean ± SEM of three independent experiments. Scale bar: 100 μm; * *p* < 0.05, ** *p* < 0.01.

To validate the target gene *p21*, the p21-3`UTR and p21-3`UTR mutant dual-luciferase vector were transfected into the porcine preadipocytes, followed by the transfection with miR-106a agomir/NC. The data indicated that miR-106a markedly suppressed the dual-luciferase activity (Figure 3B). Furthermore, RT-qPCR results showed that *p21* mRNA decreased compared with the controls (*p* < 0.01) (Figure 3C). Moreover, the p21 protein level was consistent with its mRNA level (Figure 4D,E). However, there was no significant change in the protein expression of BAMBI after the transfection of miR-106a agomir (Figure 3D,E). These results indicate that miR-106a could promote adipocyte proliferation by targeting p21 3`UTR.

### 3.4. miR-106a Promotes the Differentiation of Porcine Preadipocytes

To identify the role of miR-106a in the differentiation of subcutaneous adipocytes in porcine tissue, the miR-106a agomir or NC was transfected into the porcine preadipocytes. The results of the boron dipyrromethene (BODIPY) staining on the 8th day of differentiation indicated that miR-106a prominently increased lipid accumulation of the porcine preadipocytes. Meanwhile, the results of Oil Red O staining on the eighth day of differentiation showed a tendency similar to that of the BODIPY staining. The OD value of the stained adipocytes was also significantly increased after Oil Red O extraction (Figure 4A,B). The RT-qPCR results showed an over-expression efficiency of greater than 100 times (Figure 4C), and the expression level of the adipogenic marker genes, such as *C/EBPβ*, *PPARγ*, and *aP2*, increased compared with those of the control group at 2, 6, and 8 days of induction (Figure 4D–F). In addition, the PPARγ, aP2, and FAS protein levels were observably increased while ATGL did not significantly change at 2d or 8 d after miR-106a agomir treatment (Figure 4G,H). The western blot analysis results were coincident with the mRNA results. These results suggest that miR-106a plays a pivotal role in positively modulating the cell differentiation of porcine preadipocytes.

### 3.5. miR-106a Can Target BAMBI Instead of p21 during Porcine Preadipocytes Differentiation.

**Figure 5:**
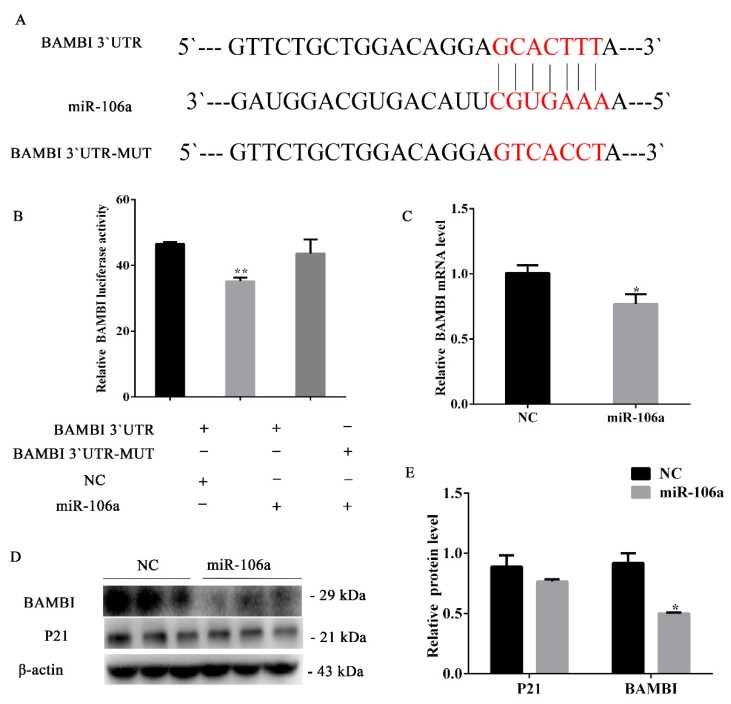
miR-106a can target *BMP and activin membrane-bound inhibitor (BAMBI)* instead of *cyclin-dependent kinase inhibitor 1A* (*p21)* during adipogenic differentiation. (**A**) The target site of miR-106a within the porcine *BAMBI* mRNA 3′UTR and mutational site of *BAMBI* 3′UTR; (**B**) the dual-luciferase assay was performed by com-transfection of the miR-106a agomir and wild-type vectors or mutant vectors. Relative luciferase activity was represented by Renilla luciferase/firefly luciferase (RLUC/FLUC); (**C**) the relative *BAMBI* mRNA expression levels after treatment with an miR-106a agomir; (**D**) western blot analysis of p21 and BAMBI protein expression after treatment with an miR-106a agomir; (**E**) the quantification of p21 and BAMBI protein expression levels. Data are representative of the mean ± standard deviation of three independent experiments. * *p* < 0.05; ** *p* < 0.01.

To investigate the mechanisms by which miR-106a accelerated porcine preadipocytes differentiation, we forecasted the target gene BAMBI of miR-106a by using TargetScan and miRTarBase (Figure 5A). Then, the psiCHECKTM-2 vector and mutation vector of the BAMBI 3′UTR were constructed (Figure 5A). Dual luciferase activity was detected during subcutaneous porcine adipocytes transfection with plasmids and the miR-106a agomir/NC. The data indicated that miR-106a markedly suppressed dual-luciferase activity (*p* < 0.01) (Figure 5B). Moreover, *BAMBI* mRNA was notably reduced on the 8th day of differentiation (*p* < 0.05) (Figure 5C). The western blot analysis results were coincident with the above described results, but there was no significant change in the protein expression of p21 (Figure 5D,E). On the whole, these findings indicate that miR-106a can alter the differentiation of porcine preadipocytes by targeting BAMBI.

## 4. Discussion

As increasingly miRNA has been found to play an important role in the development of adipose tissue, many miRNAs are regarded as potential targets or markers for metabolic diseases associated with obesity [32]. Here, the results of our analyses showed that miR-106a is highly conserved among species, suggesting that miR-106a performs similar functions among species. This is consistent with previous studies [33]. The data show that miR-106a is highly expressed in adipose tissue and functions to regulate adipocyte proliferation and adipogenesis. The overexpression of miR-106a promotes the proliferation of porcine preadipocytes. This is consistent with the studies showing that miR-106a promotes cell proliferation [34,35]. These studies found that inhibiting miR-106a can inhibited gastric cancer cell and colon cancer cell proliferation. Interestingly, the overexpression of miR-106a can also promote porcine preadipocyte differentiation. This phenomenon is consistent with studies of Li et al., who found miR-106a have dual functions in the modulation of human adipose-derived mesenchymal stem cells (hADSCs), osteoblasts, and adipocytes. The expression of miR-106a can promote adipogenesis and inhibit osteogenesis [29]. In addition to Wang et al [33]. another study found that the miR-17-92 cluster could accelerate adipocyte differentiation [30,36]. We believe that miRNAs are widely expressed in organisms, but their expression is spatially–temporally-specific. Thus, miRNAs can play different roles by targeting different genes at different time periods. Many previous studies have confirmed this viewpoint [31,37].

The cell cycle is driven by the activation of a series of cyclin proteins and CDKs along the cycle. Studies have shown that p21 can inhibit CDK2 and CKD4 activity, leading to cell arrest in the G1 phase, thereby preventing cell proliferation. Some studies indicated that miR-106a can inhibit cell proliferation [28,38]. However, most research has shown that miR-106a can promote cell proliferation. Qin et al. showed that miR-106a promotes colon cancer cell proliferation by targeting the gene of phosphate and tension homology deleted on chromosome ten (PTEN)/phosphatidyl inositol 3-kinase (PI3K)/serine-threonine kinase (AKT) signaling pathway [35]. miR-106a promotes prostate cancer cell proliferation by directly targeting PTEN in vivo and in vitro [29]. Our study showed that miR-106a promotes preadipocyte proliferation in porcine tissue by targeting *p21*. This result is consistent with previous studies showing that miR-106a can target to *p21* [39,40]. These data suggest that the overexpression of miR-106a accelerates cell proliferation by targeting *p21*. miRNAs have numerous target genes that target different genes in different cells or species [41]. Therefore, we believe that miR-106a has a different effect on proliferation because of differences in its target genes. This is also consistent with previous research [42].

BAMBI plays an important role in regulating adipogenesis. It is a transmembrane protein that encodes 260 amino acids. The BAMBI protein is a transmembrane glycoprotein and a pseudoreceptor of the TGF-β/Smad signaling pathway [43]. A large amount of data shows that BAMBI regulates many physiological and pathological process in the body [44,45,46]. In addition, BAMBI is closely related to TGF-β and Wnt/β-catenin signaling [47,48]. Studies have indicated that LINC01133 can regulate the Wnt/β-catenin signaling by adsorbing miR-106a [49]. Moreover, studies have shown that if the TGF-β signaling pathway is activated in bovine granulosa cells, miR-106a expression is increased by 2.6 times [27]. Meanwhile, it has been shown that the miR-17-92 family can inhibit the regulation of the TGF-β signaling pathway [50]. These reports are consistent with the results of miR-106a targeting *BAMBI*. In adipogenesis, it was shown that BAMBI may repress adipocytes differentiation through wnt3a, TGF-β1, and BMP4 [43]. Further studies have shown that BAMBI can inhibit porcine adipocyte differentiation by promoting the Wnt/β-catenin signaling pathway [51]. Here, we identified that BAMBI was also targeted by miR-106a in porcine preadipocytes. In addition, our results showed that the protein levels of PPARγ and aP2 were significantly increased while BAMBI was obviously decreased after overexpressing miR-106a. Therefore, we proposed that miR-106a promotes porcine adipocytes differentiation by targeting *BAMBI*.

## 5. Conclusions

**Figure 6:**
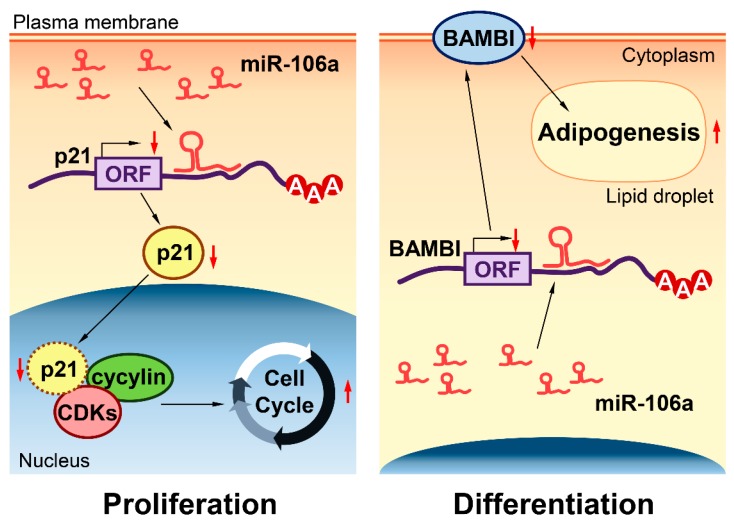
A schematic diagram of miR-106a regulation on porcine preadipocyte proliferation and differentiation. In proliferating cells (left), miR-106a can promote porcine preadipocyte proliferation by targeting *p21*. In differentiating cells (right), miR-106a can promote adipogenic differentiation by targeting *BAMBI* in porcine tissue. The red upward arrow means that this process is promoted; the red downward arrow means that this process is inhibited.

In summary, as shown in Figure 6, in a proliferating condition, miR-106a regulates the cell cycle by inhibiting p21, thereby attenuating the p21 inhibition of cyclin-CDKs and promoting porcine adipocyte proliferation. Our data show that miR-106a could promote adipocyte differentiation by targeting *BAMBI*. These findings will benefit both the control of meat quality in the animal husbandry industry and provide a new potential target for the human treatment of obesity and II type diabetes.

## Figures and Tables

**Table 1:** Primer sequences.

Gene	Primer Sequence	Length	Temp (°C)
*PPARγ*	F: AGGACTACCAAAGTGCCATCAAA R: GAGGCTTTATCCCCACAGACAC	164	60
*aP2*	F: GAGCACCATAACCTTAGATGGA R: AAATTCTGGTAGCCGTGACA	142	60
*C/EBPβ*	F: GCACAGCGACGAGTACAAGA R: TATGCTGCGTCTCCAGGTTG	98	60
*p21*	F:ACGTCTCAGGAGGACCATGT R:AGAAGATCAGCCGGCGTTTG	166	60
*BAMBI*	F:AGGACAAGGCAACAGGTATTAGC R: GAACCACAACTCTTTGGAGGAAG	96	60
*β-actin*	F: GGACTTCGAGCAGGAGATGG R: AGGAAGGAGGGCTGGAAGAG	138	60

Notes: PPARγ: peroxisome proliferator-activated receptor γ; aP2: fatty acid binding proteins; C/EBPβ: CCAAT/enhancer-binding protein β; p21: cyclin-dependent kinase inhibitor 1A; BAMBI: BMP and activin membrane-bound inhibitor.
